# Allergens Prevalence among Patients with Respiratory Allergies in Mashhad, Iran

**Published:** 2019-02

**Authors:** Payam Payandeh, Javad Fadaee, Farahzad Jabbari Azad, Mehdi Bakhshaii, Samane Sistani

**Affiliations:** 1 Department of Clinical Immunology and Allergy, Babol University of Medical Sciences, Babol, Iran,; 2 Allergy Research Center, Mashhad University of Medical Sciences, Mashhad, Iran,; 3 Sinus Endoscopic Surgery Research Center, Mashhad University of Medical Sciences, Mashhad, Iran,; 4 Department of Medical Informatics, Faculty of Medicine, Mashhad University of Medical Sciences, Mashhad, Iran

**Keywords:** Allergens, Respiratory allergies, Rhinitis, Asthma, Rhinoconjunctivitis

## Abstract

**Background:**

Respiratory allergies are among the most common allergies in the world with an increasing number of people affected in recent decades. Determination of allergens prevalence in each area is considered as the first step in prevention of allergic diseases and developing novel and more effective immunotherapies. The aim of this study was to determine the prevalence of the most common allergens among patients with respiratory allergies in Mashhad, Iran

**Materials and Methods:**

This cross sectional study included 1246 people who were referred to Allergy Clinic of Mashhad University of Medical Sciences with respiratory allergic symptoms from 2012 to 2017 in which a questionnaire containing demographic information was completed and Skin Prick Test was performed for each patient.

**Results:**

Among 1246 patients with respiratory symptoms, there were 1084 patients with allergic rhinitis (87%), 69 patients with allergic asthma (5.5%), 14 patients with allergic rhinoconjunctivitis (1.1%) and 79 patients with both allergic rhinitis and asthma (6.3%) with an overall male to female ratio of 1.18. Rhinorrhea (86.3%), sneezing (81.1%) and itchy eyes (68.4%) were the most common symptoms in patients with respiratory allergic disorders in this study and the highest rate of sensitivity was to pollens including Salsola kali (82.3%), pigweed mix (65.1%), tree mix (51.7%) and ash (49.8%), respectively.

**Conclusion:**

Generally, Salsola kali seems to be the main allergen in different respiratory allergies including allergic rhinitis, asthma and rhinoconjunctivitis in semi-arid climate of Mashhad, Iran.

## INTRODUCTION

Any substance capable of inducing production of immunoglobulin E (IgE) in a genetically predisposed individual is referred to as ‘allergen’ (1, 2). Allergens associated with animals, cockroaches, House Dust Mites (HDMs), foods, fungi, pollens, latex, and venom have been reported to be the most important factors initiating allergic responses with pollens as the most likely source of outdoor allergens and HDMs as the most common source of indoor allergens (3, 4). Pollen allergens, the male gametophyte of trees, weeds and grasses, are dust-like particles released through the pollination processes. Allergies associated with pollen particles follow a seasonal pattern, since pollens are only released in specific seasons and trigger allergic responses as they find their way through nose and bronchial airways (2, 3). Animal allergens and fungi are also considered the outdoor sources of allergens; however, some fungi species (Aspergillus species) can be found in warm and humid places inside either (5, 6). Different types of allergic diseases are also adversely affected by HDMs which usually abound indoors, in house furniture, and live in close contact with humans (2, 7, 8).

Allergic diseases have been defined as hypersensitivity disorders of the immune system as the result of allergic inflammation induced by an allergen (9–11). Identifying common allergens in each area is of great importance as avoiding allergens is the first-line prevention in controlling allergic diseases (3). Moreover, it leads to a better understanding of possible reasons for allergic symptoms in sensitive people, as well as more efficient diagnosis and treatment of a specific allergy (3, 10).

Physical examination, studying the history of patients, and some paraclinical tests usually helps with diagnosis of allergic disorders (9, 10).The Skin Prick Test (SPT) is a diagnostic test, routinely used for assessment of IgE-mediated sensitization to various allergens and is considered to provide one of the best combinations of sensitivity and specificity (9–11).The SPT is done through administration of standard commercial extracts of allergens on the forearm using a sterile lancet. The results are observed after 15 minutes in which a wheal with a 3 mm diameter greater than the negative control (saline) is considered positive (12).

Respiratory allergies are among the most frequent types of allergy in the world with an increasing incidence. According to several epidemiological studies, Prevalence of respiratory allergies is estimated between 10–30% usually with a greater affected population in urban areas rather than rural ones (13–15).

Allergic Rhinitis (AR), the most common respiratory allergy disease with an increasing prevalence (16, 17) is an inflammatory condition and is characterized by nasal symptoms such as sneezing, itchy, stuffy and runny nose (18, 19). While it is usually considered a mild health issue, it can adversely affect patients’ quality of life through direct and indirect complications and impose a heavy burden on the public health system (16, 20, 21). AR is also considered as a significant risk factor for allergic asthma, a chronic inflammatory respiratory disease characterized by reversible narrowing and hyper-responsiveness of the bronchial airway to a variety of biological and environmental stimuli with classic symptoms including wheezing, recurrent cough and dyspnea (22–24). Asthma usually coexists with rhinitis and is an important cause of chronic morbidity and mortality in the world (18, 25). Allergic Rhinoconjunctivitis (ARC) is also one of the most common chronic respiratory diseases and atopic disorders which has been less studied through the lens of epidemiology (26). Aeroallergens (airborne allergens) are important factors in respiratory allergies (3). Studies have shown that the distribution and pattern of aeroallergens significantly varies among different countries and even different regions within a country (27, 28).

Iran is a large country located between the subtropical aridity of the Arabian Desert areas and the subtropical humidity of the eastern Mediterranean area with various geo-climatic conditions in its different regions. Mashhad, the second largest and populated city of Iran, is in the Northeast of this country and has a semiarid climate described with hot summers and cold winters (11, 16). As the pattern of allergens prevalence may differ from time to time due to the environmental changes, the goal of the present study was to further investigate the most recent prevalence of aeroallergens in a larger population of patients suffering from AR, ARC, Asthma or a combination of these diseases during a 5 year period, in Mashhad, Iran using SPT in order to develop better management for respiratory allergies in different groups of people with different characteristics.

## MATERIALS AND METHODS

### Demography

A cross-sectional study was done on patients, age 1 to 74 years old, with diagnosed respiratory allergies including AR, asthma and ARC, referred to Allergy Clinics of Mashhad University of Medical Sciences during the years 2012 to 2017. In total 1246 people willing to participate in the study met the inclusion criteria; A) Diagnosis of having one or more of the above mentioned respiratory allergies by an allergy specialist based on clinical history, physical examination, specific questions and blood test if necessary, for at least 12 months in the absence of background diseases; B) No treatment affecting the SPT for at least one week prior to the test. Accordingly, patients with underlying diseases such as cold, flu, chronic sinusitis or other infective causes of rhinitis were excluded from the study, so were those taking drugs affecting the SPT including antihistamines and steroids. A questionnaire was completed by each patient in order to collect the demographic data such as age, gender, disease pattern (perennial or intermittent), family history of atopic disease, living place (rural or urban) and physical examination. The Ethics Committee of the Immunology Research Centre, Mashhad, Iran approved the study protocol and all patients officially consented to participate in this study by signing an agreement before the test.

### Skin prick test

SPT was carried out for all patients using common allergen extracts following international guidelines, in which histamine chloride and normal saline were considered as positive and negative controls respectively. The standardized allergens extracts (Greer, USA) were put on both fore-arms using sterile lancets. Test results were read after 15 minutes through measuring the wheal diameters formed due to skin reaction. SPT was considered positive if the wheal diameter was at least 3 mm larger than the negative control. Outdoor aeroallergen extracts including Russian thistle (Salsola kali), pigweed mix, ash (Fraxinus excelsior), Birch tree, grass mix, tree mix, and indoor extracts such as Dermatophagoides farina (D. farina), Dermatophagoides pteronyssinus (D. pteronyssinus), cockroach, feather, and indoor and outdoor allergens such as Alternaria alternate, Aspergillus mix, Cladosporium and Candida albicans were used in this test. Selection of allergens was based on regional conditions and relevant studies.

### Statistics

Statistical analysis, descriptive statistics and the Chi-square test were done using SPSS 22. P values smaller than 0.05 (P<0.05) were considered statistically significant.

## RESULTS

### Demography

Out of 1445 patients who showed positive SPT to at least one of the aeroallergens, 1246 people (676 men and 570 women) were affected by one or a combination of aforementioned respiratory allergic diseases and were selected for further studies including 1084 AR (87%), 69 asthma (5.5%),14 ARC (1.1%) and 79 AR and asthma (6.3%) with a greater number of male patients in all four groups (overall male/female ratio was 1.18). However, there was no statistically significant relationship between gender and having a respiratory allergy, neither with a specific disease. Table 1 shows the demographic characteristics of the participant patients.

**Table 1.** Frequency and the percentage of demographic characteristics in general and each allergic disease

**Characteristics**		**AR**	**AR+AA**	**AA**	**ARC**	**Overall**
**Gender**	Male	586(54.1%)	48(60.8%)	37(53.6%)	9(64.3%)	586(54.1%)
	Female	498(45.9%)	31(39.2%)	32(46.4%)	5(35.7%)	498(45.9%)
**Disease Pattern**	Perennial	613(56.5%)	45(57%)	37(53.6%)	7(50%)	702(56.3%)
	Intermittent	471(43.5%)	34(43%)	32(46.4%)	7(50%)	544(43.7%)
**Family History**	Positive	634(58.5%)	43(54.4%)	41(59.4%)	9(64.3%)	728^*^(58.3%)
	Negative	442(40.8%)	36(45.6%)	28(40.6%)	5(35.7%)	511(41%)
**House**	Old	338(31.2%	28(35.4% )	19(27.5%)	3(21.4%)	388^**^(31.1%)
	New	744(68.6%)	51(64.6%)	50(72.5%)	11(78.6%)	856(68.7%)
**Living Place**	Urban	873(80.5%)	62(78.55)	54(78.3%)	12(85.7%)	1001(80.3%)
	Rural	211(19.5%)	17(21.5%)	15(21.7%)	2(14.3%)	245(19.7%)
**Area**	Indoor	794(73.2%)	67(84.8%)	60(87%)	12(85.7%)	933(74.9%)
	Outdoor	149(13.7%)	8(10.1%)	8(11.6%)	2(14.2%)	167(13.4%)
	I/O	141(13%)	4(5.1%)	1(1.4%)	0(0%)	146(11.7%)
**Exacerbation Month**	January	663(61.2%)	50(63.3%)	42(60.9%)	7(50%)	762(61.2%)
	February	663(61.2%)	50(63.3%)	43(62.3%)	7(50%)	763(61.2%)
	March	681(62.85)	51(64.6%)	42(60.9%)	7(50%)	781(62.7%)
	April	802(74%)	58(73.4%)	51(73.9%)	11(78.6%)	922(74%)
	May	854(78.8%)	59(74.7%)	52(75.4%)	11(78.6%)	976(78.3%)
	June	895(82.6%)	58(73.4%)	51(73.9%)	12(85.7%)	1016(81.5%)
	July	914(84.3%)	58(73.4%)	52(75.4%)	11(78.6%)	1035(83.1%)
	August	893(82.4%)	57(72.2%)	52(75.4%)	10(71.4%)	1012(81.2%)
	September	872(80.4%)	56(70.9%)	50(72.5%)	10(71.4%)	988(79.3%)
	October	729(67.3%)	55(69.6%)	43(62.35)	7(50%)	834(66.9%)
	November	682(62.9%)	52(65.8%)	43(62.3%)	7(50%)	784(62.9%)
	December	671(61.9%)	51(64.6%)	42(60.9%)	7(50%)	771(61.9%)

I/O: Showing symptoms both indoors and outdoors,

*: There were 8 cases with missing information,

**: There were 2 cases with missing information

The perennial pattern of the disease was prevalent in overall (56.3%) and also in all groups (56.5% in AR, 53.6% in asthma, 57% in AA+AR and 50% in ARC). However, equal number of patients with ARC suffer from perennial and intermittent pattern of the disease (50%). Generally, 728 (58.3%) patients mentioned a family history of atopic disorders. No significant relationship was found between the type of the respiratory disease and family history. Although 856 (68.7%) patients had moved to a new house in recent years, no statistically significant difference was found between living in an old or a new house and having any kinds of studied allergic diseases. The prevalence of the diseases was significantly more among patients living in the city rather than those living in rural areas with P value of 0.02. Totally, 1001 (80.3%) patients lived in urban areas and 245 (19.7%) lived in rural and suburban parts. Among all, 933 (74.9%) patients reported exacerbation of their symptoms in closed space (indoors), while 167 (13.4%) reported to be suffering more in open space (outdoors) and 146 (11.7%) in both closed and open areas. The most severe form of the diseases was observed in July, June and August (summer in general) according to the complaint of 1035 (83.1%), 1016 (81.5%) and 1012 (81.2%) people, respectively. Prevalence of allergic symptoms both generally and in regards with each disease is shown in table 2.

**Table 2.** Distribution of allergic symptoms in different respiratory diseases

**Symptoms**	**AR**	**AR+AA**	**AA**	**ARC**	**Overall**
**Rhinorrhea**	942(86.9%)	67(84.8%)	53(76.8%)	13(92.9%)	1075(86.3%)
**Sneezing**	890(82.1%)	62(78.5%)	47(68.1%)	12(85.7%)	1011(81.1%)
**Itchy eyes**	749(69.1%)	49(62%)	42(60.9%)	12(85.7%)	852(68.4%)
**Itchy nose**	717(66.1%)	50(63.3%)	42(60.9%)	9(64.3%)	818(65.7%)
**Nasal Congestion**	659(60.8%)	49(62%)	43(62.3%)	7(50%)	758(60.8%)
**Itchy throat**	581(53.6%)	42(53.2%)	27(39.1%)	7(50%)	657(52.7%)
**Red eyes**	478(44.1%)	36(45.6%)	22(31.9%)	5(35.7%)	541(43.45)
**Itchy Skin**	369(34%)	30(38%)	20(29%)	2(14.3%)	421(33.85)
**Sore throat**	353(32.6%)	32(40.5%)	26(37.7%)	8(57.1%)	417(33.5%)
**Sinus infection**	298(27.5%)	26(32.9%)	19(27.5%)	4(28.6%)	347(28.7%)
**Burning eyes**	298(27.5%)	22(27.8%)	11(15.9%)	3(21.4%)	334(26.8%)
**Sputum**	277(25.6%)	28(35.4%)	16(23.2%)	3(21.4%)	324(26%)
**Shortness of Breath**	252(23.3%)	31(39.2%)	19(27.5%)	1(7.1%)	303(24.3%)
**Swollen eyelids**	221(20.4%)	14(17.7%)	15(21.7%)	3(21.4%)	253(20.3%)
**Wheezing**	173(16%)	30(38%)	18(26.1%)	1(7.1%)	222(17.8%)
**Red spot**	163(15%)	14(17.7%)	12(17.4%)	14(100%)	189(15.2%)
**Anorexia**	154(14.2%)	18(22.8%)	6(8.75)	3(21.4%)	181(14.5%)
**Heartburn**	122(11.3%)	15(19%)	7(10.1%)	2(14.3%)	146(11.7%)
**Regurgitation**	106(9.8%)	12(15.2%)	8(11.6%)	3(21.4%)	129(10.4%)
**Nausea**	96(8.9%)	12(15.2%)	6(8.7%)	1(7.1%)	115(9.2%)
**Chest pain**	82(7.6%)	16(20.3%)	4(5.8%)	1(7.1%)	103(8.3%)
**Stomachache**	81(7.5%)	10(12.7%)	6(8.7%)	1(7.1%)	98(7.9%)
**Vomit**	52(4.85)	9(11.4%)	4(5.6%)	1(7.1%)	66(5.3%)
**Diarrhea**	46(4.2%)	6(7.6%)	6(8.7%)	1(7.1%)	59(4.7%)
**Blisters**	35(3.2%)	3(3.8%)	3(4.3%)	0(0%)	41(3.3%)

The most prevalent symptom was rhinorrhea, observed in 1075 86.3%) patients, followed by sneezing (81.1%) and itchy eyes (68.4%) as the second and third ranks, respectively. While, blisters was the rarest symptom, only observed in 41 (3.3%) people out of the total number of 1246. Rhinorrhea and sneezing were also the most prevalent symptom among people suffering from AR (86.9 and 82.1%), AA (76.85 and 68.1%) and both (84.8% and 78.5%). While in people with ARC, red spot was observed in all 14 (100%) patients as the most frequent symptom, followed by rhinorrhea (92.9%) as the second prevalent symptom and sneezing (85.7%) and itchy eyes (85.7%) in the third place.

### Skin prick test

Sensitivity to Salsola Kali, pigweed mix, tree mix, ash and grass mix were observed in 1025 (82.3%), 811 (65.1%), 644 (51.7%), 621 (49.8%), 570 (45.7%) patients, respectively, which were the most prevalent allergens. None of the patients were allergic to Cladosporium (0%) and only 1 patient (0.1%) showed allergic response to feather, which makes it the second least prevalent allergen. Sensitivity to Salsola Kali was also the most frequent in patients of each disease group (84.7% in AR, 87.5% in AA, 57.1% in ARC and 53.6% in AA+AR).

**Figure 1.**
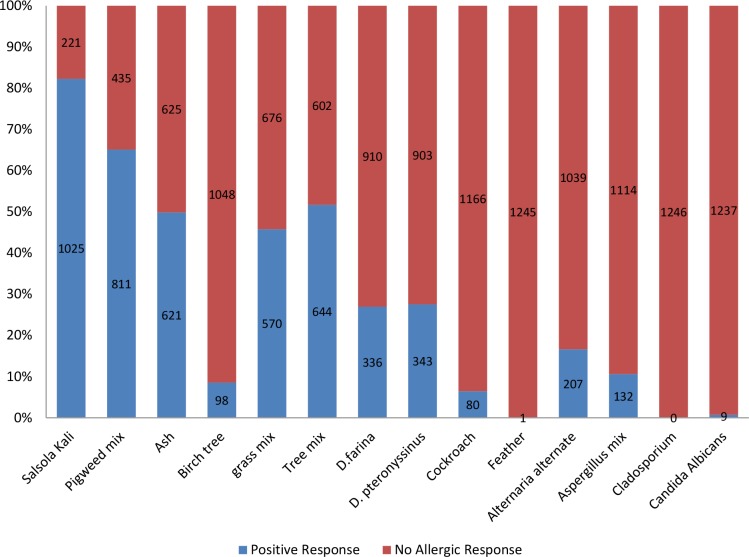
The allergens sensitivity distribution in all 1246 patients

Pigweed mix was the second prevalent allergen among patients with AR (67.6%), ARC (50% = prevalence of D. farina) and AA+AR (59.5%), while D. pteronyssinus was the second prevalent allergen in patients with asthma (46.4%). D. pteronyssinus and D. farina were the most prevalent indoor allergens in total (27.5% and 27%) and also among patients with asthma (46.4% and 34.8%) and patients with both asthma and AR (43% and 31.6%). With a reverse order, they were also the most common indoor allergens among patients with AR (D. farina in 25.8% and D. pteronyssinus in 25.1% of patients with AR) and ARC (D. farina in 50% and D. pteronyssinus in 35.7% of patients with ARC). The general prevalence of the allergens is shown in figure 1 and their prevalence according to each respiratory disease is shown in table 3.

**Table 3.** Allergens sensitivity distribution in allergic respiratory diseases

**Allergens**		**AR**	**AR+AA**	**AA**	**ARC**	**Overall**	**P Value**
**Outdoor Allergens**	**Salsola kali**	918(84.7%)	62(78.5%)	37(53.6%)	8(57.1%)	1025(82.3%)	<0.001
	**Pigweed mix**	733(67.6%)	47(59.5%)	24(34.8%)	7(50%)	811(65.1%)	<0.001
	**Tree mix**	573(52.9%)	40(50.6%)	27(39.1%)	4(28.6%)	644(51.7%)	<0.001
	**Ash**	576(53.1%)	28(35.4%)	14(20.3%)	3(21.4%)	621(49.8%)	<0.001
	**Grass mix**	522(48.2%)	32(40.5%)	13(18.8%)	3(21.4%)	570(45.7%)	<0.001
	**Birch tree**	92(8.5%)	5(6.3%)	1(1.4%)	0(0%)	98(7.9%)	0.02
**Indoor Allergens**	**D. pteronyssinus**	272(25.1%)	34(43%)	32(46.4%)	5(35.7%)	343(27.5%)	0.003
	**D.farina**	280(25.8%)	25(31.6%)	24(34.8%)	7(50%)	336(27%)	0.1
	**Alternaria alternate**	175(16.1%)	17(21.5%)	13(18.8%)	2(14.3%)	207(16.6%)	0.07
	**Aspergillus mix**	110(10.1%)	12(15.2%)	10(14.5%)	0(0%)	132(10.6%)	0.14
	**Cockroach**	76(7%)	2(2.5%)	2(2.9%)	0(0%)	80(6.4%)	0.02
	**Candida Albicans**	7(0.6%)	0(0%)	2(2.9%)	0(0%)	9(0.7%)	0.59
	**Feather**	1(0.1%)	0(0%)	0(0%)	0(0%)	1(0.1%)	0.97
	**Cladosporium**	0(0%)	0(0%)	0(0%)	0(0%)	0(0%)	-

There is a significant difference in sensitivity to Salsola kali, ash, grass mix, pigweed mix, tree mix (p< 0.001), D. pteronyssinus, (p=0.003), Birch tree (p=0.02) and cockroach (p=0.2) in different respiratory diseases with a higher prevalence among people with AR.

## DISCUSSION

This study included people with symptoms of AR, allergic asthma, ARC and a combination of AR and allergic asthma which equals 86.2% of all 1445 patients who were referred to allergy clinic of Mashhad University of Medical Sciences and had positive SPT. Prevalence of AR (87%) was considerably higher than other respiratory allergies. Generally pollens were the most common allergens with Salsola kali (82.3%) as the most prevalent allergen among all 4 disease groups of this study which is in accordance with the studies by Mahram et al. and Mahboubi Oskouei et al. who both reported Salsola kali as the most common allergen with a percentage of 50.2 and 58.2%, respectively (3, 11). However, these findings are in contrast with the study by Ghaffari et al. in Sari, Iran in which mites (D. farina, D. pteronyssinus and cockroach) were reported as the most common allergens (18). This contrast could be due to major climatic difference between two cities. Unlike Mashhad, Sari has a humid climate which makes the indoor areas more suitable for mites to thrive. Pollens were also reported as the most common allergens in neighboring countries such as Jordan (29) and Kuwait (30). On the contrary, HDM were reported as the most prevalent allergens in Thailand (31), Singapore (32) and Mexico city (33). Sensitivity to pollens seems to be increasing among allergic patients in Mashhad, comparing to the similar prior study done by Mahboubi Oskouei et al. during 2010–2014 in the same area in which 50.2, 36.7, 29.1, 21.6 and 19.5% were reported as the frequency percentage of sensitivity to Salsola kali, ash, grass mix, tree mix and pigweed mix, respectively (11). However, a considerably higher prevalence of these allergens were observed in the present study where (82.3%), (65.1%), (51.7%), (49.8%), (45.7%) of patients were sensitive to Salsola Kali, pigweed mix, tree mix, ash and grass mix as the first to fifth most prevalent allergens, respectively. While pollens generally remained the most common allergens with Salsola kali as the first rank, the order of other allergens in this group regarding their prevalence has slightly changed during the time within the same area of Iran (Mashhad), which both could be due to the continuous urban development and its effects on vegetation pattern.

Rhinorrhea and sneezing were the first and second common symptoms among patients with AR, asthma and AR-asthma. Amizadeh et al. also reported rhinorrhea as the most common symptoms among patients with AR (76.6%) (34). Rhinorrhea and sneezing followed by pruritus and congestion were also reported as the most common symptoms of AR in the research done by Ghaffari et al. in Sari (18) which is the same as the results of the present study. They also reported cough, dyspnea and wheezing as the most common symptoms in patients with asthma (18) which is in contrast with present study. Although there was a higher prevalence of allergic diseases among patients with a family history of atopic disorder, no statistically significant correlation was found between these variables in the present study. There was also no significant correlation between gender and sensitivity to allergens which is in accordance with the studies done by Kashef et al. in Shiraz (35) and Farrokhi et al. in Bushehr (9). A significant relationship was found between AR and sensitivity to Salsola kali, ash, grass mix, pigweed mix, tree mix, D. pteronyssinus, birch tree and cockroach. A similar correlation between AR and ash, grass mix, pigweed mix, tree mix had been reported in Mashhad (11).

## CONCLUSION

In conclusion, determination of allergen pattern and the rate of allergic patients’ sensitivity to each allergen in each area are of great importance in controlling and treatment of allergic disorders. Salsola kali, pigweed mix, tree mix and ash were the most provocative allergens in Mashhad, Iran.
